# Effect of Social Media on Patient's Perception of Dental Aesthetics in Saudi Arabia

**DOI:** 10.1155/2022/4794497

**Published:** 2022-02-27

**Authors:** Khadijah M. Baik, Ghazal Anbar, Abeer Alshaikh, Arwa Banjar

**Affiliations:** ^1^Oral and Maxillofacial Prosthodontics Department, Faculty of Dentistry, King Abdulaziz University, Jeddah, Saudi Arabia; ^2^BDS, Faculty of Dentistry, King Abdulaziz University, Jeddah, Saudi Arabia; ^3^Department of Periodontology, Faculty of Dentistry, King Abdulaziz University, Jeddah, Saudi Arabia

## Abstract

**Introduction:**

Social media became an influential tool that affects people's way of communication and became a significant source of information for the society. The study aimed to evaluate the impact of SM on patients seeking aesthetic dental treatment. *Methodology*. The study employed a large-scale online survey of 1940 patients attending and/or seeking dental treatment at KAUFD and Jeddah private clinics. The targeted age of participants ranged from 18 years and above. The study data were collected using a three-part questionnaire.

**Results:**

More than half of the patients were females in both groups (52.7%). The majority of patients used SM for communication and entertainment purposes. It was also found that the most popular platform used by patients was Snapchat (71.1%), followed by Instagram (66.9%). A lot of patients did not like their teeth appearance (38.5%). Moreover, patients preferred to have “bleaching” as an aesthetic treatment to improve their smiles (63.8%).

**Conclusion:**

The impact of SM on Saudi Arabian citizens and Saudi Arabia residents can be considered as high. Patients are influenced by SM applications and are seeking aesthetic treatment as an outcome. It is the responsibility of dentists to educate patients about the best treatment options.

## 1. Introduction

Social media (SM) is a powerful tool that affects not only communication but also relationships among people. SM nowadays is called a social-cultural agent of change that uses information and affects the provider-patient interaction. SM has begun to spread across the medical field, and nowadays, patients take it as a source of information. It is considered a useful tool for the dentist and the patient [1]. Even though patients use SM in their personal lives, little is known about their attitudes and expectations toward using SM for professional interactions. SM marketing is a more useful marketing technique compared to traditional marketing. A study conducted in Riyadh among dentists reported that Twitter was the most commonly used platform, where 43% reported that they use SM for educational purposes. It was also reported that 62% and 68% used SM to promote their dental practice and broadcast treatment outcomes, respectively [2]. Smile aesthetics can provide valuable insights into posttreatment satisfaction and can predict the patients' goals for receiving treatment [3].

SM has a strong impact on people's lives, and it is similar in Saudi Arabia and the Arabian Gulf region [4]. Patients look for dental information, follow and connect with dentists, and write about their experience through SM. In Saudi Arabia, it is reported that the majority of participants preferred receiving health-related information from trusted official sources [5]. The outcomes of dental aesthetic treatment have a huge psychological impact on the patients, where poor outcomes may damage the dentist-patient relationship. Dentists should know how to get patients' attention to dental problems and their treatments by posting cases on SM in a simple manner that the people easily understand. This will encourage patients to seek dental treatment and raise their awareness about the actual treatments and their benefits. In addition, patients could use SM to have background information about dental treatments and their indications, especially if it is from an aesthetic view. Also, they need to know how to communicate with their dentists and ask more about the aesthetic treatments before any interventions such as an “online consultation,” which will make patients more comfortable and confident with their dental staff. There is rapid growth and influence of SM on patients' behaviors, but until now, there is not sufficient evidence about the effect of SM on demand for aesthetic dental treatment in Saudi Arabia. Thus, this study aim is to check the impact of SM effect on patients seeking aesthetic dental treatment.

## 2. Materials and Methods

A cross-sectional analytical study was carried out since September 2019 among patients seeking dental treatment in Jeddah city, Saudi Arabia. The questionnaire was distributed to patients seeking dental treatment at King Abdulaziz University Faculty of Dentistry (KAUFD) dental hospital as well as private clinics. The study sample consisted of 1940 random patients (50% from KAUFD and 50% from private clinics), and the time for completion of the said study was 56 days (8 weeks). The inclusion criteria were patients who ranged from 18 to older than 45 years and came to KAUFD and private clinics for dental treatments. All nationalities and socioeconomic status levels will be included, and geriatric (over 60 years old) and pediatric (below 18 years old) patients will be excluded. Human subject protection was taken into consideration by explaining the purpose and the procedure of the current study to participating patients. Consequently, ethical approval was released to begin this research (REC no. 155-11-19) from the Research Ethics Committee, Faculty of Dentistry, King Abdulaziz University.

For test-retest reliability, 10 individuals were asked to complete the survey, and then they repeated the survey a second time after a one-week period. A pretested questionnaire was used for data collection that had three parts: (a) sociodemographic data, (b) patients' satisfaction of their current dentition from the aesthetic view, and (c) questions regarding SM applications and their usage related to dental purposes. There were a total of 28 questions, with an estimated time to complete the survey to be around 5–10 minutes. Consent e-forms were then provided and distributed electronically to all participants, using “SurveyMonkey” program. Questionnaires were distributed and collected either in person using electronic tablets or were sent via WhatsApp application messages. Participants had the option of either filling the survey in Arabic or English language.

### 2.1. Statistical Analysis

A minimum sample size of 1123 was calculated assuming a proportion of 0.5 and desired precision of the estimate was 0.05 and at a 95% confidence level. Sample size estimating software used was nMaster 2.0. (CMC, Vellore). The Statistical Package for Social Sciences, version 23 (SPSS Inc., Chicago, IL, USA), was used for the data analysis. Categorical variables were presented as numbers and percentages. Continuous variables were measured using mean and standard deviation. Pearson's chi-square test was used to find an association between categorical variables. A *p* value less than 0.05 was considered statistically significant.

## 3. Results

**Figure 1:**
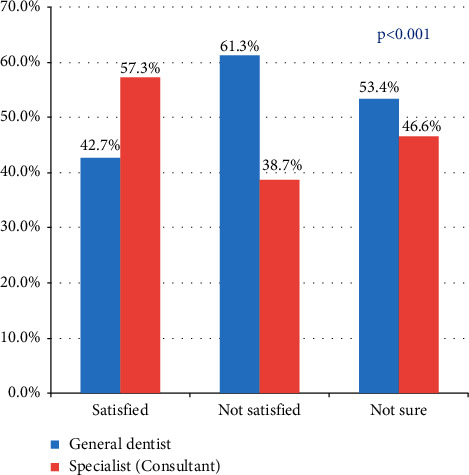
Relationship between satisfication about treatment done and the person performed it (*n* = 740).

The analysis included a total response from 1940 participants from KAUFD clinical (50%) and private dental clinics (50%). The sociodemographic characteristics showed that 52.7% were females, 32% belonged to 18–25 years, 57% were married, 77.6% were Saudi citizens, 49.2% had a graduate level of education, and 43.6% belonged to medical sectors. When we assessed the satisfaction about the shape and color of their natural teeth, 38.5% reported that they did not like them. Among this, 29.5% (*n* = 220) reported that this dislike was after following a dental/dentist account or page in SM. The most common aesthetic dental treatment preferred to be done is bleaching (63.8%) followed by orthodontics (39.4%), crowns (30%), and veneers (21.2%). The most common reason to choose the above aesthetic dental treatment was recommendation from family and friends (76.2%), whereas only 12.7% mentioned it as “SM” impact, and 5.1% did this due to “followers and like” for the dental or dentist's account or page in SM. It was reported by 51.6% that a ‘specialist or consultant' did the treatment, and 59.5% were satisfied with this treatment. When we analyzed the relationship between these two, it was found that participants were comparatively more satisfied with the treatment that is performed by a specialist or consultants (57.3%) than that done by the general dentist (42.7%), which showed a statistical significance (*p* < 0.001) (Figure 1).

The most common aspect of the teeth that participants were dissatisfied with was chipped or broken teeth (50%), followed by tooth shape (38.7%), gum health (37.1%), and tooth color (33.9%), where 91.9% (*n* = 114) reported that they would attempt for another treatment for its correction (Table 1).

It was believed by a majority of the participants (79.7%) that dental prosthesis is not lifelong. When we asked participants' perception about the life expectancy of veneers and crowns, 47.8% believed that it is less than 10 years, whereas only 23% mentioned it as more than 10 years. The practices related to SM usage showed 92.1% used social media (SM), where Snapchat (71.1%) was the commonly used one and 86.7% of them used SM daily. The most common purpose for its usage was for ‘communication' (77.6%), and it was found that only 36.6% followed a dental or dentist's account on SM (Table 2).

The usage of SM was comparatively more seen in females (94.1%), participants aged 18–25 years (98.1%), who were single (97.1%), participants who had educational qualification at the graduate level (94.5%), and whose profession was engineering (95.6%) than others (*p* < 0.001). Their usage was comparatively low in participants who had a monthly income of more than 15,000 SAR (84.8%) than others (*p* < 0.001) (Table 3).

When we assessed the pattern of SM usage between two genders, participants who followed dentists'/dental accounts in SM were comparatively females (43%, *p* < 0.001), aged 18–25 years (47.4%, *p* < 0.001), those who had bachelor degree (38.6%, *p* = 0.040), and those who belonged to the medical sector (41.2%) more than others. When asked whether the dental content in SM is accurate or not, participants aged 26−35 years (11.1%, *p* = 0.015), non-Saudi nationalities (10.9%, *p* < 0.001), those who had diplomas (14.9% *p* < 0.001), and those from the educational sector (10.8%, *p* < 0.001) comparatively more agreed that it is accurate than others. When participants were asked whether the photos of the dental treatment outcome (before/after) encourage patients to seek treatment, females (57%, *p* = 0.006), participants aged 26−35 years (60.1%, *p* < 0.001), non-Saudi nationals (56.7%, *p* = 0.043), participants with the education of high school level (58.3%,*p* = 0.001), and those who belonged to the medical sector (56%, *p* = 0.019) were the ones who agreed comparatively more to this than others (Table 4).

## 4. Discussion

SM has become a very influential tool that affects our communication and relationships with people, and like every other tool, it has its pros and cons. SM nowadays is called a “social-cultural agent of change” that uses data to alter the provider-patient interaction. The use of SM in the medical field has grown exponentially and has become one of the main sources of information for the patient. In fact, it is considered a useful tool for the dentist and the patient; even though the patients use SM in their personal lives, we still do not know enough about their attitudes and what they expect from its interactions [6]. The current study shows that SM marketing for dentists is more useful compared to traditional marketing practices. Studies show that most of the dentists recommended the usage of SM by their colleagues because it had a significant effect on their career, and the majority had a positive effect on their dental practice [3, 7]. As far as the patients are concerned, there are many patients who are already using different SM to connect with their dentists. In our study, younger age groups were more frequent users of SM, and the majority was females because it affected their choices, especially in the aesthetic field and different kinds of treatments. At the present time, aesthetic dental treatment has become a priority for most of the population, but not all patients know what the causes might be and parameters that affect the appearance of the smile, dental arch characteristics, dentogingival, and dentolabial [8]. Smile aesthetics have a huge effect on patient satisfaction and patient expectations in the ongoing treatment [9]. According to research conducted among dental students, females were more concerned and critical about dental aesthetics, with hypodontia being the most distracting feature of a smile when assessing its beauty, followed by a gingival smile, a reversed occlusal plane, and dental crowding [10]. Protrusion of teeth, poorly aligned teeth, carious and discolored restorations, and fractured teeth all influence the dental appearance of the patients [10, 11]. A cross-sectional study was done in one of the dental schools in the United Kingdom to examine SM use, perceptions, and attitudes towards SM and the survey in the study reported that most of the participants were using SM at least once a week; more than one application and a majority had an idea about how can SM affect their dental practice positively [12]. Another study done in Saudi Arabia reported that 98% of the participants had at least one account on SM, 81% used it daily, and 66% of patients used it as a source of information [13]. Dentists may be unable to take advantage of SM activities that benefit both dentists and patients due to a lack of understanding of patients' attitudes toward utilizing SM for dental treatment purposes. A study done in New Zealand among general dental practitioners showed that television affects the population in seeking for various aesthetic dental procedures, mainly bleaching and veneers [14]. Dental practitioners may also face problems managing their professional image and relationships with patients as a result of widespread and rapid access to information [15, 16]. Patients and dentists may face additional problems that threaten their privacy as a result of their use of SM. It is reported that dentists do not well understand concepts, methods, and processes linked to SM communication [17]. Some concerns, such as after-clinic care and dental anxiety, can be addressed via social networks. Dental anxiety is a serious issue in dentistry, which often causes depression, sleep disorders, an unwillingness to establish intimate interpersonal relationships, and difficulty at workplaces [18, 19]. Wider qualitative research may allow us to analyze successful cases of SM usage in dentistry, which would provide some specific suggestions for dental professionals. It is the responsibility of dentists to educate patients about the best treatment option.

## 5. Conclusion

The study confirmed that SM has recently become a priority in the Saudi population, and it developed very quickly. Also, it becomes an important tool in most of our daily life needs such as communication, education, entertainment, and healthcare. Therefore, the impact of SM on Saudi Arabian citizens and Saudi Arabia residents can be considered as high. Patients are influenced by the SM applications and are seeking aesthetic treatment as an outcome.

## Figures and Tables

**Table 1:** Baseline characteristics of the participants.

		*N*	%
Gender	Male	918	47.3
	Female	1022	52.7

Age	18–25	620	32.0
	26–35	412	21.2
	36–45	430	22.2
	>45	478	24.6

Social status	Single	770	39.7
	Married	1106	57.0
	Divorced	64	3.3

Nationality	Saudi	1506	77.6
	Non-Saudi	434	22.4

Educational qualification	No primary education	10	.5
	High school	688	35.5
	Diploma	158	8.1
	Bachelor degree	954	49.2
	Masters/PhD	130	6.7

Job sectors	Medical	846	43.6
	Engineering	364	18.8
	Educational	212	10.9
	Fashion and business	202	10.4
	Accounting	164	8.5
	Others	152	7.8

Income (SAR)	<5000 SAR	376	19.4
	5000–15000 SAR	516	26.6
	>15000	330	17.0
	No salary	718	37.0

**Table 2:** Satisfaction and attitudes about dental aesthetics.

		*n*	%
Likes the shape/color of your current teeth (*n* = 1940)	Agree	578	29.8
	Disagree	746	38.5
	Not sure	616	31.8

If does not like the teeth, it was after following dental accounts/public figures in social media (*n* = 746)	Agree	220	29.5
	Disagree	292	39.1
	Not sure	234	31.4

Aesthetic dental treatment likes to be done for your teeth (*n* = 746)	Orthodontic	294	39.4
	Bleaching	476	63.8
	Crown	224	30.0
	Veneers	158	21.2
	Crown lengthening surgery	70	9.4
	Filler injection for the face	38	5.1
	Botox injection	28	3.8
	Others	24	3.2

Did dental consultation for the aesthetic problem (*n* = 1940)	Yes	950	49.0
	No	990	51.0

Did aesthetic dental treatment in the past 5 years (*n* = 1940)	Yes	740	38.1
	No	1200	61.9

Type of aesthetic dental treatment done (*n* = 740)	Bleaching	34	4.6
	Veneers	236	31.9
	Crowns	292	39.5
	Crown lengthening surgery	30	4.1
	Orthodontics	240	32.4
	Filler injection for the face	10	1.4
	Botox injection	14	1.9

Reasons made to choose the above aesthetic dental treatment (*n* = 740)	Recommendation from family and friends	564	76.2
	Social media	94	12.7
	Special offers	114	15.4
	Followers and likes	38	5.1
	Certificates and awards of the dentist	66	8.9
	Quality of before/after pictures	78	10.5

Person who did aesthetic dental treatment (*n* = 740)	General dentist	358	48.4
	Specialist/consultant	382	51.6

You are satisfied with your dental treatment you had done (*n* = 740)	Satisfied	440	59.5
	Not satisfied	124	16.8
	Not sure	176	23.8

Aspect of teeth that did not give satisfaction (*n* = 124)	Tooth shape	48	38.7
	Tooth color	42	33.9
	Chipped or broken teeth	62	50.0
	Bulky teeth	20	16.1
	Gum health	46	37.1
	Oral smell	34	27.4
	Effect on facial profile	12	9.7
	Others		

Attempt another treatment to correct it (*n* = 124)	Yes	114	91.9
	No	10	8.1

**Table 3:** Perception about dental prosthesis.

	Responses	*N*	%
Think that the dental prosthesis is for life	Yes	372	19.2
	No	1546	79.7
	Not sure/do not know	22	1.1

Life expectancy of veneers and crowns	<10 years	928	47.8
	10 years	566	29.2
	>10 years	446	23.0

**Table 4:** Practices related to social media use.

		*N*	%
Use any social media app	Yes	1786	92.1
	No	154	7.9

Type of social media apps used (*n* = 1786)	Instagram	1194	66.9
	Snapchat	1270	71.1
	Facebook	456	25.5
	Twitter	1014	56.8

Frequency of its usage (*n* = 1786)	Daily	1548	86.7
	Monthly	30	1.7
	Rarely	140	7.8
	Weekly	68	3.8

Purpose of its usage	Communication	1386	77.6
	Entertainment	1274	71.3
	Education	1082	60.6
	Advertisement	338	18.9

Follow any dental or/and dentist account	Yes	654	36.6
	No	1132	63.4

All dental contents present in social media are accurate	Agree	134	7.5
	Disagree	784	43.9
	Not sure	868	48.6

Photos of the dental treatment outcome (before/after) encourage patients to seek treatment	Agree	966	54.1
	Disagree	194	10.9
	Not sure	626	35.1

## Data Availability

The data presented in this study are available within the article.
